# Factors predicting discharge outcomes of sepsis patients admitted to intensive care unit in a major tertiary care hospital: A retrospective study from Palestine

**DOI:** 10.1371/journal.pgph.0005643

**Published:** 2025-12-19

**Authors:** Elias Ashqar, Aidah Alkaissi, Osama Ateeq, Ramzi Shawahna

**Affiliations:** 1 Critical Care Nursing Program, Faculty of Graduate Studies, An-Najah National University, Nablus, Palestine; 2 An-Najah National University Hospital, An-Najah National University, Nablus, Palestine; 3 Department of Nursing and Midwifery, Faculty of Nursing, An-Najah National University, Nablus, Palestine; 4 Department of Physiology, Pharmacology, and Toxicology, Faculty of Medicine and Allied Health Sciences, An-Najah National University, Nablus, Palestine; 5 Clinical Research Center, An-Najah National University, Nablus, Palestine; University of Colorado Anschutz Medical Campus: University of Colorado - Anschutz Medical Campus, UNITED STATES OF AMERICA

## Abstract

Sepsis remains a leading cause of morbidity and mortality among critically ill patients, particularly in resource‐limited settings where diagnostic capacity and therapeutic options are constrained. In this study, we aimed to identify clinical, laboratory, and treatment factors that independently predict in‐hospital mortality among adult sepsis patients admitted to a tertiary care intensive care unit (ICU) in the West Bank of Palestine. We conducted a retrospective cohort study of 326 adult patients (aged 18–80 years) admitted with sepsis to the medical ICU of a major tertiary referral hospital between January 2018 and December 2023. In‐hospital mortality was 41.4% (n = 135). Predictors of mortality were assessed using a multivariable logistic regression model. Multivariable logistic regression identified advancing age (OR 1.03 per year; 95% CI: 1.01-1.06; p = 0.010), cardiovascular disease (OR 2.66; 95% CI: 1.17-6.04; p = 0.020), elevated heart rate (OR 1.03 per beat/min; 95% CI: 1.01-1.04; p < 0.001), reduced urine output (OR 1.00 per mL; 95% CI: 1.00-1.00; p = 0.035), elevated serum lactate (OR 1.15 per mmol/L; 95% CI: 1.01-1.30; p = 0.037), prolonged ventilator days (OR 1.15 per day; 95% CI: 1.09-1.21; p < 0.001), lower PaO_2_/FiO_2_ ratio (OR 1.00 per unit; 95% CI: 1.00-1.00; p = 0.006), and shorter ICU length of stay (OR 0.91 per day; 95% CI: 0.87-0.96; p < 0.001) as independent predictors of in‑hospital mortality. These findings highlight the prognostic importance of simple bedside measures, core laboratory indices, and markers of illness trajectory. Together, they form a pragmatic panel of universally available variables that reliably stratify mortality risk among septic ICU patients in Palestine. Embedding these predictors into admission checklists and electronic health record alerts could strengthen early risk recognition, guide triage decisions, and optimize allocation of scarce resources in resource‑limited settings.

## Introduction

Sepsis ranks among the leading causes of morbidity and mortality in hospitalized patients, arising when a dysregulated host response to infection triggers life-threatening organ [1,2]. Approximately one in five patients presenting with infection in the emergency department deteriorates within 48 hours, underscoring how rapidly sepsis can progress to severe organ failure and death [3]. When sepsis advances to septic shock, it may precipitate acute kidney injury, acute respiratory distress syndrome, myocardial depression, hepatic dysfunction, and neuromuscular complications, each further increasing mortality risk [4]. These serious sequelae highlight the imperative for early recognition and aggressive management to improve survival. It has been estimated that the global in-hospital mortality rates for sepsis range from 20% to over 50%, depending on the number and severity of organ failures and the infection source [3,5,6]. As the leading cause of death in critical care, sepsis places an urgent demand on intensive organ support and prolonged, resource-intensive treatment in the intensive care unit (ICU) [3,7].

Extensive research in well-resourced settings has identified a range of clinical and laboratory markers that predict sepsis mortality. Readily available bedside measures, systolic blood pressure, heart rate, and Glasgow Coma Scale (GCS) score [8], alongside biomarkers such as serum lactate and albumin, demonstrate consistent correlations with survival outcomes [9,10]. In addition, validated scoring systems, Acute Physiology and Chronic Health Evaluation II (APACHE II) [11], Sequential Organ Failure Assessment (SOFA) [12], GCS, and Simplified Acute Physiology Score II (SAPS II) [13], improve prognostic precision by stratifying patients according to mortality risk at ICU admission [14,15]. Despite substantial progress in elucidating sepsis pathophysiology and prognostic markers, the majority of available evidence originates from high‑income countries, where patient demographics, microbial landscapes, and critical‑care resources differ markedly from those in resource‑limited settings such as Palestine. Several studies from low‑ and middle‑income countries (LMICs) have explored the epidemiology and outcomes of sepsis, and collectively they reveal substantial heterogeneity in mortality predictors across diverse clinical and geographic contexts. For example, reports from Africa emphasize the impact of delayed presentation, limited microbiological capacity, and high burdens of HIV and tuberculosis, whereas Latin American studies underscore resource constraints and antimicrobial resistance patterns distinct from those in high‑income settings [16–21]. Previous studies from Palestine have shown that the burden of sepsis is exacerbated by contextual constraints. In particular, the local microbial ecology is characterized by a high prevalence of multidrug‑resistant Gram‑negative organisms, which may profoundly affect therapeutic effectiveness and clinical outcomes [22,23].

In resource‑limited healthcare systems, the burden of sepsis is compounded by contextual challenges. Constraints in diagnostic capacity, including limited access to blood cultures and advanced microbiological assays, delay pathogen identification and contribute to both under‑ and over‑diagnosis of sepsis [24–26]. Antimicrobial stewardship practices are variably implemented, reflecting differences in institutional resources, guideline adherence, and prescriber behavior, which in turn lead to inconsistent antibiotic exposure and increased multidrug resistance [27,28]. Furthermore, the high prevalence of comorbidities such as diabetes and malignancy modifies sepsis presentation and prognosis, with diabetes linked to infection susceptibility and acute kidney injury, and malignancy associated with immunosuppression and poor outcomes [29–31].

The Palestinian healthcare system is characterized by limited access to advanced diagnostic modalities, restricted intensive care unit capacity, inconsistently implemented antibiotic stewardship, and constrained therapeutic options, all of which accentuate disparities in sepsis management compared with resource‑rich environments [32,33]. The high prevalence of comorbid conditions, particularly diabetes mellitus and malignancy, further modifies the clinical presentation and prognosis of sepsis in our population. Taken together, these contextual challenges underscore the necessity of identifying mortality predictors that are specifically adapted to our region, rather than relying on models developed in high‑income settings.

A prior study conducted in Palestine described the epidemiology of sepsis syndrome among ICU patients, focusing primarily on incidence and descriptive outcomes [22]. However, bedside variables, laboratory parameters, and severity scores were not systematically evaluated as independent predictors of mortality. Therefore, this study was conducted to extend evidence by identifying context‑specific predictors of in-hospital mortality among patients admitted with sepsis, thereby addressing a critical gap in understanding sepsis outcomes in Palestinian ICUs. The findings of this study propose to inform earlier recognition, guide targeted interventions, and optimize allocation of scarce critical-care resources within a resource-limited healthcare environment.

## Methods

### Study design

We conducted a retrospective cohort study, identifying a defined group of patients from existing medical records and evaluating their in‑hospital outcomes through systematic chart review. In this study, all adults admitted with sepsis to the ICU between January 2018 and December 2023 were assembled as the cohort, and their clinical, physiological, and biochemical data were abstracted retrospectively. Records were accessed and data were collected in the period between January 1, 2024 and April 30, 2024. This design is particularly well suited to our aims because it enables efficient evaluation of multiple predictors against an endpoint (in-hospital mortality) without the time and expense of prospective follow-up. Similar sepsis investigations have successfully used retrospective cohorts to delineate risk factors and validate prognostic scores [9,10,14,34]. The study is reported in adherence to the Strengthening the Reporting of Observational Studies in Epidemiology (STROBE) Statement (S1 Table).

### Setting

The study was conducted at the major tertiary referral hospital in the West Bank of Palestine. As the region’s primary referral hospital, it serves a catchment of eleven governorates and manages a high volume of complex medical and surgical cases. Its comprehensive electronic health record system and centralized ICU databases allowed us to capture a diverse patient population and a wide array of variables, ranging from vital signs and laboratory values to severity scores, thereby ensuring our findings are both robust and generalizable within resource‑limited critical‑care settings. In the West Bank of Palestine, governmental hospitals, United Nations Relief and Works Agency for Palestine Refugees in the Near East (UNRWA)‑affiliated hospitals, and privately owned hospitals employ electronic health record systems [35]. Similarly, many hospitals in LMICs have largely employ electronic medical record systems. It is important to note that employing electronic health record systems alone does not eliminate systemic resource constraints. Restricted ICU capacity, limited availability of advanced diagnostics, constrained therapeutic options, and variability in antimicrobial stewardship remain defining features of critical‑care practice in these settings [32,33].

### Participants

The source population comprised all adult patients (aged 18–80 years) admitted with a clinical diagnosis of sepsis to the medical ICU at the major academic tertiary referral center between January 2018 and December 2023. Cases were identified via the hospital’s electronic health record system and confirmation against standardized clinical criteria. Patients older than 80 years, those with ICU stays under 24 hours, and those with incomplete medical records were excluded. All included individuals were followed from ICU admission until in-hospital death or discharge, with dates and outcomes fully captured through the electronic health record.

### Sample size

We calculated our minimum sample size at 294 patients based on an anticipated in-ICU sepsis mortality of 25.8% [34], a two-sided α of 0.05, and a 5% margin of error, then inflated this by 10% to allow for potential data loss. From 434 charts retrieved, we excluded 49 patients older than 80 years, to minimize confounding by advanced age and multiple comorbidities, especially since APACHE II and SAPS II scores assign disproportionate weight to age > 80 years, 6 patients with ICU stays under 24 hours, and 53 patients with incomplete demographic, vital-sign, or clinical data. The remaining 326 patients met our predefined criteria and comprised the final cohort for analysis.

### Variables

Sepsis was defined according to the Third International Consensus Definitions for Sepsis and Septic Shock (Sepsis‑3) [36]. Specifically, sepsis was characterized as life‑threatening organ dysfunction caused by a dysregulated host response to infection, operationalized as an increase of ≥ 2 points in the SOFA score in the presence of suspected or confirmed infection. Septic shock was defined as a subset of sepsis with persistent hypotension requiring vasopressor support to maintain a mean arterial pressure of ≥ 65 mmHg and a serum lactate level > 2 mmol/L despite adequate fluid resuscitation. These standardized criteria were applied to confirm diagnoses across all included patients.

Our primary outcome was in-hospital mortality, dichotomized as discharge alive versus death during the ICU stay. Mortality was assessed exclusively within the ICU. Patients who died during their ICU stay were classified as “dead,” whereas those discharged alive from the ICU were classified as “alive.” Outcomes following ICU discharge, including deaths occurring before hospital discharge or after transfer to other facilities, were not captured, as the scope of this study was limited to ICU‑level mortality. Discharge from the ICU was determined according to standardized clinical criteria. Patients were considered eligible for transfer once they were alert and conscious, with stable vital signs maintained without pharmacological support (e.g., vasopressors) or supplemental oxygen. Laboratory parameters were required to have returned to values within the normal range, and all clinical indicators of sepsis, including fever, leukocytosis, and other infection‑related abnormalities, had to be resolved. Clearance of blood cultures and normalization of severity scores (e.g., GCS, SOFA) were also taken into account to ensure that patients no longer met criteria for critical illness prior to ICU discharge.

Exposures and predictors encompassed demographic factors (age, sex) and preexisting comorbidities (hypertension, diabetes mellitus, cardiovascular disease, chronic renal or hepatic disease, malignancy). Systemic inflammatory response syndrome status was considered, defined by standard thresholds for temperature, heart rate, respiratory rate, and white blood cell count [37]. However, in line with the recommendations of the Surviving Sepsis Campaign Guidelines [38], which no longer endorse systemic inflammatory response syndrome as a diagnostic criterion for sepsis, this variable was excluded from the final analysis to ensure methodological consistency with contemporary sepsis definitions.

Physiological data were recorded on ICU admission, either immediately or after any urgent resuscitation within the first hour, and included the modified early warning score (MEWS), initial systolic and diastolic blood pressures, mean arterial pressure, heart rate, temperature, respiratory rate, and the GCS. Norepinephrine was administered as the first‑line vasopressor in patients with severe, life‑threatening hypotension. The therapeutic goal was to maintain a mean arterial pressure of at least 65 mmHg, consistent with international sepsis management guidelines, in order to ensure adequate perfusion of vital organs. Patients with a GCS score of ≤ 8 were considered unresponsive.

Laboratory predictors comprised 24-hour urine output, C-reactive protein (CRP), serum bicarbonate, platelet count, total bilirubin, arterial partial pressure of oxygen, hematocrit, white blood cell count, serum creatinine, blood urea nitrogen, serum sodium, serum potassium, arterial pH, serum albumin, and serum lactate. We also calculated validated severity indices, APACHE II, SOFA, and SAPS II, each with its predicted mortality probability. Tracheal aspirate culture was defined as a lower respiratory tract specimen obtained via sterile endotracheal suction in intubated patients; specimens were processed in the microbiology laboratory using standard quantitative culture techniques, and a culture was considered positive when a potentially pathogenic organism grew at ≥ 10^5^ colony forming units (CFU)/mL. This variable complemented other culture findings, blood, urine, and sputum, by specifically assessing ventilated patients for lower airway infection and guiding antibiotic selection. Source of infection was obtained from the medical records of the patients. Treatment variables included antibiotic administration (e.g., levofloxacin, tigecycline), vasoactive support (norepinephrine, epinephrine, dopamine, dobutamine), mechanical ventilation modality (invasive versus noninvasive), fraction of inspired oxygen, ventilator days, and partial pressure of oxygen/fraction of inspired oxygen ratio (PaO_2_/FiO_2_) ratio. Age, sex, and comorbidity burden were considered potential confounders, and we explored effect modification by key conditions such as malignancy through interaction analyses.

### Data sources and collection

All data were extracted from the electronic health record system and centralized ICU databases of the major academic tertiary referral center, which integrated demographic details, comorbidities, vital signs, laboratory results, treatment interventions, and discharge outcomes. Two registered nurses with ICU experience (EA and OA) performed the chart abstraction using a pre-piloted, standardized electronic form that was developed specifically for this study. Prior to data collection, they received training led by the study supervisors (AA and RS) to ensure consistent interpretation of variable definitions and uniform data-entry procedures.

Vital signs (blood pressure, heart rate, respiratory rate, temperature, MEWS, GCS) were recorded by bedside nurses as the first measurements on ICU admission (within the first hour) and captured in the electronic health record system. Laboratory measurements (e.g., CRP, bicarbonate, bilirubin, electrolytes, lactate) were obtained via the hospital’s central laboratory under standardized operating procedures. Culture results (blood, urine, sputum, tracheal aspirate) reflected quantitative thresholds in the microbiology database. Severity scores (APACHE II, SOFA, SAPS II) were retrospectively calculated by the trained abstractors (EA and OA) using published scoring algorithms applied to the recorded clinical and laboratory values, ensuring consistency and comparability across all patients.

Respiratory specimens were collected according to the mode of ventilation. Tracheal aspirate samples were obtained from patients undergoing invasive mechanical ventilation, where the presence of an endotracheal tube allowed direct sampling from the lower airways using a suction catheter. Sputum samples were collected from patients receiving non‑invasive mechanical ventilation, as well as from those not on mechanical ventilation, provided they were able to produce an adequate deep‑cough specimen. Sources of infection were confirmed using a combination of microbiological, radiological, and clinical criteria. While blood and other cultures were obtained, many patients had received antibiotics prior to ICU admission, which likely reduced culture positivity. In cases where cultures were negative, infection sites were identified through radiological imaging (e.g., chest X‑ray, ultrasound) and clinical assessment by the treating physicians, based on standardized diagnostic criteria. Blood cultures and other microbiological specimens were collected immediately upon admission to the ICU, prior to initiation of antibiotics within the ICU. However, data on antibiotic administration before ICU admission were not available. As a result, we were unable to assess the impact of prior antibiotic exposure on culture yield or mortality outcomes.

Malignancy was differentiated according to SAPS II requirements as either hematological or solid tumor malignancy, and the score was calculated accordingly. For the purposes of our outcome analysis, however, all patients with malignancy were grouped under a single category. This approach was chosen to evaluate the overall impact of malignancy on survival and mortality among patients admitted with sepsis, rather than to compare outcomes across specific subtypes. The data collection form is provided in S2 Table.

To ensure accuracy and consistency, each abstractor double-checked the entered data; discrepancies were verified and resolved through discussion and consensus during weekly review meetings. Supervisors additionally cross-checked the data against source records to confirm data fidelity. Because all patients were managed within the same ICU environment and captured via a single electronic health record system, measurement methods and data sources were fully comparable across the cohort.

Incomplete records were defined as patient charts with missing data on one or more key predictor variables required for univariate and multivariate analyses. These variables included demographic characteristics, baseline comorbidities, vital signs, laboratory parameters, and severity scores (APACHE II, SAPS II, SOFA). Records with systematic gaps, such as absent laboratory panels or missing severity score documentation, were excluded to ensure analytic consistency. By restricting the dataset to complete records, we minimized bias introduced by non‑random missingness and preserved the validity of our outcome analyses.

### Ethical consideration

This study was conducted in accordance with the Declaration of Helsinki and received approval from the Institutional Review Board (IRB) of An-Najah National University. Given its retrospective design, the IRB waived the requirement for written informed consent. All patient data were deidentified and stored in a secure, access-restricted database, with audit trails and encryption in place to safeguard confidentiality.

### Statistical methods

All statistical analyses were performed using IBM SPSS Statistics for Windows, Version 25.0 (IBM Corp., Armonk, NY, USA). Descriptive statistics summarized categorical variables as counts (n) and percentages (%) and continuous variables as medians with interquartile ranges (IQR: Q1-Q3). We assessed normality using the Shapiro-Wilk test. For bivariate comparisons, we used Chi-square or Fisher’s exact tests for categorical variables and Mann-Whitney U tests for non-normal continuous variables. Statistical significance was defined as two-tailed p-value < 0.05.

To address potential multicollinearity among predictor variables (e.g., overlapping measures across APACHE II, SOFA, and SAPS II scores, or between GCS and responsiveness categories), we conducted diagnostic testing using multiple linear regression. Variance inflation factors (VIF) and tolerance statistics were calculated for all candidate variables. Variables with VIF > 5 or tolerance < 0.2 were considered to exhibit problematic collinearity and were excluded from further inclusion in the multivariate logistic regression model. To identify independent predictors of in-hospital mortality, we performed multivariable logistic regression after removing collinear variables. This approach ensured that the final model was both statistically robust and clinically meaningful. Model calibration was assessed using the Hosmer-Lemeshow goodness‑of‑fit test. Explained variance was quantified by Cox & Snell R^2^ and Nagelkerke R^2^. Discrimination was evaluated by the C‑statistic (area under the ROC curve). Effect sizes were reported as adjusted odds ratios (OR) with 95% confidence intervals (95% CI).

## Results

### Demographic and baseline variables of the patients

We included 326 adult patients admitted to the ICU with a sepsis diagnosis. The median age was 58.0 years [IQR: 45.0-68.0], and 58.9% (n = 192) were male. On admission, 15 patients (4.6%) could communicate verbally, and only 6 (1.8%) were able to communicate pain. Respiratory tract infections predominated, accounting for 30.7% of cases, followed by urinary tract infections at 21.5%. Bloodstream infections represented 15.6%, and wound or pressure‐ulcer sources contributed 10.7% (S1 Fig). Detailed demographic and baseline characteristics are presented in Table 1.

**Table 1:** Demographic and baseline variables of the patients (*n = 326*).

Variable	Median [Q1, Q3] or n (%)
Age (Years)	58.0 [45.0, 68.0]
**Sex**	
Male, n (%)	192 (58.9)
Female, n (%)	134 (41.1)
**Comorbidities**	
Hypertension, n (%)	132 (40.5)
Diabetes mellitus, n (%)	108 (33.1)
Malignancy, n (%)	191 (58.6)
Renal disease, n (%)	120 (36.8)
Liver disease, n (%)	29 (8.9)
Cardiovascular disease, n (%)	85 (26.1)
Systemic inflammatory response syndrome, n (%)	232 (71.2)
**Neurologic status**	
Unresponsive (GCS ≤ 8)	105 (32.2)
Verbal responsiveness (able to speak)	15 (4.6)
Pain responsiveness (able to report pain)	6 (1.8)

Q1: lower quartile, Q3: upper quartile

### Vital signs and laboratory findings of the patients

Patients exhibited significant deteriorated pathophysiologic parameters on ICU admission. Tachypnea was common, with a median respiratory rate of 23.0 breaths/min [IQR: 19.0-26.0], and the median MEWS was 4.0 [IQR: 3.0-6.0], indicating moderate acuity. Laboratory values reflected an acute inflammatory response as indicated by elevated CRP values (median 156.0 mg/L [IQR: 72.8-260.0]). Hematologic compromise was also evident, with a median platelet count of 134.0 K/µL [IQR: 41.5-261.3] and a median hematocrit of 26.8% [IQR: 23.0-30.7]. Detailed vital signs and laboratory findings are presented in Table 2. Although the overall median GCS score on admission was 14.0 [IQR 3.0-15.0], 105 patients (32.2%) presented with GCS ≤ 8 and were therefore classified as unresponsive. As S2 Fig shows, the GCS distribution is strongly bimodal: one peak at 15 (fully alert) and another at 3 (deep coma). The lower bound of the IQR (3) reflects the unresponsive subgroup, while the upper quartile (14–15) reflects those with near-normal consciousness.

**Table 2:** Vital signs and laboratory findings of the patients (*n = 326*).

Variable	Median [Q1, Q3]
**Vital signs**	
Systolic blood pressure (mmHg)	110.0 [100.0, 120.0]
Diastolic blood pressure (mmHg)	60.0 [50.0, 70.0]
Mean arterial pressure (mmHg)	76.0 [67.0, 85.0]
Heart rate (beats/min)	100.0 [85.0, 120.0]
Temperature (°C)	37.0 [36.5, 37.9]
Respiratory rate (breaths/min)	23.0 [19.0, 26.0]
MEWS	4.0 [3.0, 6.0]
GCS	14.0 [3.0, 15.0]
**Laboratory findings**	
Urine output over 24 hours (mL/day)	1615.0 [890.0, 2470.0]
CRP (mg/L)	156.0 [72.8, 260.0]
Bicarbonate (mmol/L)	21.5 [18.0, 25.0]
Platelet count (K/uL)	134.0 [41.5, 261.3]
Total serum bilirubin (mg/dL)	0.8 [0.4, 2.3]
Partial pressure of oxygen (mmHg)	98.5 [80.0, 120.0]
Hematocrit (%)	26.8 [23.0, 30.7]
White blood cells (K/uL)	10.5 [5.8, 17.9]
Serum creatinine (mg/dL)	1.2 [0.7, 3.1]
Blood urea nitrogen (mg/dL)	30.0 [17.0, 56.3]
Serum sodium (mmol/L)	138.0 [135.0, 143.0]
Serum potassium (mmol/L)	4.1 [3.7, 4.6]
pH	7.4 [7.3, 7.4]
Serum albumin (g/dL)	2.7 [2.4, 3.1]
Serum lactate (mmol/L)	2.0 [1.3, 3.5]

CRP: C-reactive protein, GCS: Glasgow Coma Scale, K: 1,000, MEWS: Modified early warning score, Q1: lower quartile, Q3: upper quartile

### Culture findings, treatment, ventilation, and discharge outcomes

Culture results showed the highest positivity rates in blood (19.3%) and urine (18.1%) specimens. Empiric antibiotic regimens most commonly included vancomycin (62.3%) and meropenem (56.7%), reflecting coverage for Gram-positive and broad-spectrum pathogens. Vasoactive support was required in most patients, with 72.7% receiving norepinephrine. Mechanical ventilation was also frequent: 40.8% underwent invasive ventilation and 57.1% received noninvasive support. Severity scores underscored the acute illness burden, with a median APACHE II of 19.0 [IQR: 15.0-27.0] and a median SOFA of 9.0 [IQR: 6.0-12.0]. The median length of hospital stay was 8.0 days [IQR: 4.0-16.0], and the overall in-hospital mortality was 41.4%. Detailed treatment and outcome variables are presented in S3 Table.

### Associations between discharge outcomes and the variables of the patients

Non-survivors exhibited significantly higher rates of malignancy (p < 0.001), systemic inflammatory response syndrome (p = 0.027), and unresponsiveness on admission (p < 0.001). They also demonstrated greater physiologic deterioration, with higher MEWS (p < 0.001), heart rate (p < 0.001), CRP (p = 0.035), bilirubin (p < 0.001), lactate (p < 0.001), FiO_2_ requirements (p < 0.001), and severity scores, APACHE II, SOFA, and SAPS II (all p < 0.001). Conversely, non-survivors had lower systolic blood pressure (p < 0.001), mean arterial pressure (p = 0.039), GCS (p < 0.001), 24-hour urine output (p = 0.042), platelet count (p = 0.004), albumin (p < 0.001), pH (p = 0.003), and PaO_2_/FiO_2_ ratios (p < 0.001). Invasive mechanical ventilation was more frequent among non-survivors (p < 0.001). The significant associations are shown in S4 Table.

### Factors predicting mortality outcomes among sepsis patients admitted to the ICU

Multivariable logistic regression (Table 3) identified several independent predictors of in-hospital mortality. Advancing age was associated with increased odds of death (OR 1.03; 95% CI: 1.01-1.06; p = 0.010), underscoring the vulnerability of older patients to sepsis-related complications. Cardiovascular disease conferred a markedly elevated risk (OR 2.66; 95% CI: 1.17-6.04; p = 0.020), likely reflecting the compounded burden of impaired perfusion and organ reserve. Tachycardia on admission was a robust predictor (OR 1.03 per beat/min; 95% CI: 1.01-1.04; p < 0.001), consistent with its role as a surrogate marker of systemic stress and hemodynamic instability. Reduced urine output over 24 hours was significantly associated with mortality (OR 1.00 per mL; 95% CI: 1.00-1.00; p = 0.035), reflecting early renal dysfunction and impaired clearance. Elevated partial pressure of oxygen (PaO_2_) was paradoxically associated with increased mortality (OR 1.01; 95% CI: 1.00-1.03; p = 0.018), potentially indicating underlying ventilation-perfusion mismatch or oxygenation failure despite supplemental support. Serum lactate levels were positively correlated with mortality risk (OR 1.15 per mmol/L; 95% CI: 1.01-1.30; p = 0.037), reaffirming lactate as a reliable biomarker of tissue hypoperfusion and metabolic derangement. Ventilator days were also predictive (OR 1.15 per day; 95% CI: 1.08-1.21; p < 0.001), suggesting that prolonged mechanical ventilation may reflect both disease severity and iatrogenic complications. Notably, lower PaO_2_/FiO_2_ ratios (OR 0.99 per unit; 95% CI: 0.98-1.00; p = 0.006) and shorter ICU length of stay (OR 0.91 per day; 95% CI: 0.87-0.96; p < 0.001) were independently associated with mortality, indicating that patients who succumbed tended to deteriorate rapidly with refractory hypoxemia. Collectively, these findings delineate a constellation of physiologic, biochemical, and treatment-related variables that independently stratify mortality risk in septic ICU patients, offering a pragmatic framework for early prognostication and resource prioritization in constrained healthcare settings. Hosmer-Lemeshow goodness‑of‑fit test (χ^2^ = 10.322, df = 8, p = 0.243) indicated adequate fit. Cox & Snell R^2^ = 0.382 and Nagelkerke R^2^ = 0.515 indicated acceptable variance. C‑statistic yielded an AUC of 0.813, consistent with good predictive performance. The overall classification accuracy of the final model was 81.3%, with sensitivity 71.9% and specificity 88.0%.

**Table 3:** Factors predicting mortality outcomes among sepsis patients admitted to the ICU.

					95% CI for OR	
Variable	B	SE	p-value	OR	Lower	Upper
Age	0.031	0.012	**0.010**	1.03	1.01	1.06
Sex	0.404	0.328	0.218	1.50	0.79	2.85
Hypertension	0.191	0.376	0.612	1.21	0.58	2.53
Diabetes mellitus	0.386	0.388	0.320	1.47	0.69	3.15
Renal disease	0.112	0.478	0.815	1.12	0.44	2.86
Liver disease	-0.556	0.557	0.318	0.57	0.19	1.71
Cardiovascular disease	0.978	0.419	**0.020**	2.66	1.17	6.05
Heart rate	0.027	0.008	**< 0.001**	1.03	1.01	1.04
Temperature	-0.233	0.164	0.156	0.79	0.57	1.09
Respiratory rate	0.039	0.032	0.216	1.04	0.98	1.11
Urine output/24 h	0.000	0.000	**0.035**	1.00	1.00	1.00
C-reactive protein	0.002	0.001	0.120	1.00	1.00	1.00
Bicarbonate	0.072	0.041	0.075	1.08	0.99	1.16
Platelet count	-0.002	0.001	0.066	1.00	1.00	1.00
Total serum bilirubin	0.012	0.042	0.784	1.01	0.93	1.10
Partial pressure of oxygen	0.014	0.006	**0.018**	1.01	1.00	1.03
Hematocrit	-0.020	0.029	0.482	0.98	0.93	1.04
White blood cells	0.011	0.008	0.169	1.01	1.00	1.03
Serum creatinine	-0.025	0.087	0.777	0.98	0.82	1.16
Blood urea nitrogen	0.009	0.009	0.328	1.01	0.99	1.03
Serum sodium	0.043	0.026	0.101	1.04	0.99	1.10
Serum potassium	-0.014	0.243	0.955	0.99	0.61	1.59
pH	-0.424	0.640	0.508	0.65	0.19	2.30
Serum albumin	-0.394	0.288	0.172	0.67	0.38	1.19
Serum lactate	0.135	0.065	**0.037**	1.15	1.01	1.30
Vasoactive	-0.688	0.381	0.071	0.50	0.24	1.06
Ventilator days	0.136	0.028	**0.000**	1.15	1.08	1.21
PaO2/FiO_2_	-0.005	0.002	**0.006**	1.00	0.99	1.00
Length of stay	-0.093	0.025	**0.000**	0.91	0.87	0.96

CI: confidence interval, OR: odds ratio, PaO_2_/FiO_2_: Partial pressure of oxygen/fraction of inspired oxygen ratio, SE: standard error, statistically significant p-values are in boldface

## Discussion

This study provides the first comprehensive evaluation of sepsis mortality predictors in a Palestinian tertiary‑care ICU, revealing a constellation of physiologic, biochemical, and treatment‑trajectory variables that independently stratify risk. Advancing age and pre‑existing cardiovascular disease emerged as strong determinants of death, reflecting diminished physiologic reserve and the compounded burden of circulatory compromise in septic patients [6,39,40]. These findings underscore the importance of incorporating comorbidity profiles into admission risk assessments, particularly in populations with high burdens of chronic disease.

Markers of early physiologic instability, tachycardia and reduced urine output, were independently associated with mortality. Tachycardia reflects sympathetic activation and systemic stress, and has been consistently linked to adverse outcomes in sepsis [9,10]. Oliguria, in turn, signals early renal dysfunction, a complication repeatedly associated with sepsis‑related acute kidney injury and poor prognosis [4,31]. These simple bedside measures, obtainable within the first hour of ICU admission, provide clinicians with rapid, actionable indicators of patients at heightened risk.

Biochemical markers of hypoperfusion and oxygenation deficits added further granularity to risk stratification. Elevated serum lactate, a well‑established surrogate of tissue hypoxia, was strongly predictive of death, reinforcing its central role in sepsis resuscitation strategies [14,38]. Lower PaO_2_/FiO_2_ ratios characterized non-survivors, consistent with the prognostic significance of refractory hypoxemia in sepsis‑related acute respiratory distress syndrome [12,15]. Together, these indices highlight the value of integrating biochemical and respiratory parameters into admission protocols to capture the severity of systemic derangements.

Indicators of illness trajectory, prolonged ventilator days and shorter ICU length of stay among non-survivors, further distinguished outcomes. Extended mechanical ventilation likely reflects both underlying disease severity and exposure to ventilator‑associated complications [41,42]. Conversely, rapid deterioration leading to early death underscores the urgency of timely recognition and aggressive intervention [34]. These findings suggest that both prolonged dependency and precipitous decline are critical pathways to mortality, each requiring tailored clinical strategies.

26 When contrasted with high‑income settings, several similarities and differences emerge. Predictors such as elevated lactate, impaired oxygenation indices, and prolonged ventilator dependency have consistently been reported across diverse cohorts, underscoring their universal prognostic value [12,14,38]. However, markers that often retain independent significance in high‑income studies, such as albumin, CRP, and systolic blood pressure, did not remain predictive in our adjusted model, likely reflecting the confounding influence of chronic comorbidities, nutritional heterogeneity, and limited monitoring capacity in our population [9,10,43]. Moreover, treatment‑related variables such as antibiotic choice and ventilation modality, which are sometimes emphasized in high-income countries cohorts, were excluded here due to collinearity and potential selection bias. These contrasts highlight the importance of regionally validated models, while some physiologic and biochemical markers are broadly generalizable, others may lose discriminatory power in resource‑limited environments, reinforcing the need for tailored prognostic frameworks in LMICs.

From a systems perspective, the prominence of simple physiologic and laboratory measures suggests that effective risk stratification does not require advanced diagnostics, but rather consistent documentation and rapid interpretation of universally available variables. Embedding these predictors into electronic health record alerts or triage checklists could enable frontline clinicians to identify high‑risk patients early, even in overcrowded ICUs. For example, integration of urine output, lactate, and PaO_2_/FiO_2_ ratio into automated electronic health record alerts could provide real‑time risk stratification, while bedside triage checklists incorporating heart rate and comorbidity burden would allow nurses and junior physicians to escalate care promptly. At the policy level, investment in strengthening routine monitoring capacity, such as reliable urine output tracking, lactate assays, and oxygenation indices, may yield greater survival benefits than focusing exclusively on high‑cost technologies. By aligning clinical practice and health‑system planning around predictors that are both validated and feasible in our setting, Palestinian ICUs can move toward more equitable and evidence‑based sepsis care.

### Strengths of the study

This study has several notable strengths. First, it represents the first rigorous evaluation of sepsis mortality predictors in a Palestinian tertiary‑care ICU, thereby closing a critical regional knowledge gap and providing evidence tailored to a resource‑limited setting. Second, our five‑year, single‑center cohort of 326 patients afforded substantial statistical power to detect clinically meaningful associations, while maintaining methodological consistency across admissions. Third, we captured a broad spectrum of variables, ranging from simple bedside measures (e.g., heart rate, urine output, oxygenation indices) to detailed laboratory markers (e.g., lactate, electrolytes, platelet count) and validated severity scores (APACHE II, SOFA, SAPS II), enabling nuanced multivariable modeling. Fourth, data abstraction was conducted by trained ICU nurses using a pre‑piloted electronic form, with inter‑rater reliability checks and weekly consensus reviews, ensuring high data fidelity. Fifth, anchoring our analyses in a centralized electronic health record system minimized missing data and standardized measurement protocols across all admissions. Finally, by focusing on predictors that are universally available and reproducible, our findings provide actionable insights for strengthening triage protocols, early warning systems, and capacity planning in similar resource‑constrained environments.

### Limitations of the study

Despite the notable strengths, this study has some limitations. First, the retrospective, single-center design limits causal inference. However, because our hospital serves as the primary referral center for all eleven West Bank governorates, the findings retain strong regional relevance. Second, our study excluded patients older than 80 years and those with ICU stays shorter than 24 hours. While these criteria were applied to ensure methodological consistency, particularly in relation to APACHE II and SAPS II weighting, they inevitably constrain the external validity of our findings. Specifically, the exclusion of very elderly patients limits the applicability of our results to populations with higher age distributions, where sepsis outcomes may differ substantially. Similarly, omitting patients with brief ICU stays may underestimate early mortality or rapid recovery patterns, thereby narrowing the generalizability of our predictive model to longer‑stay cohorts. These restrictions should be considered when extrapolating our findings to broader ICU populations. Third, although a number of covariates, including malignancy, systemic inflammatory response syndrome criteria, neurologic status (e.g., GCS responsiveness), systolic and mean arterial pressures, hypoalbuminemia, thrombocytopenia, hypernatremia, and treatment‑related variables such as antibiotic choice, vasopressor support, and non‑invasive ventilation, were associated with mortality in univariate analyses, they did not retain independent significance in the final multivariable model after adjustment for collinearity and confounding. Their exclusion reflects both statistical considerations and the risk of overfitting, but it also underscores the possibility that these variables may still contribute to mortality risk through complex interactions not fully captured in our dataset. For example, the apparent survival advantage of non‑invasive ventilation may partly reflect selection bias, as less critically ill patients are more likely to receive this modality, whereas more unstable patients are intubated directly. Similarly, the observed associations with antibiotic omission (e.g., levofloxacin) could be confounded by local prescribing practices and resistance patterns, which were not systematically measured through antibiogram data. Laboratory markers such as albumin, platelets, and sodium may have been influenced by chronic comorbidities and nutritional heterogeneity, limiting their discriminatory power in adjusted models. These considerations highlight the need for cautious interpretation of univariate associations and reinforce the importance of prospective studies that incorporate pathogen‑specific data, standardized stewardship protocols, and more granular measures of illness severity. It is important to note that the practical usability of laboratory predictors such as lactate and albumin was not limited in our study setting, as the tertiary referral hospital maintains rapid laboratory response times. However, in hospitals where turnaround times are longer, the real‑time applicability of these predictors may be constrained. Delays in reporting could reduce their utility for early risk stratification and timely clinical decision‑making, underscoring the need for workflow adaptations or point‑of‑care testing in such environments. Fourth, we did not systematically record the primary infection focus, which precludes source-specific risk stratification. Our comprehensive culture data and systemic inflammatory response syndrome documentation partially offset this by capturing the overall microbiologic and inflammatory milieu. Fifth, important process variables, exact timing of antibiotic administration, fluid resuscitation volumes, and detailed resistance patterns, were not available. These represent logical targets for future prospective research rather than fundamental flaws in our prognostic modeling. While our analysis focused on in‑hospital mortality as a binary endpoint, future work should incorporate time‑to‑event analyses to provide a more nuanced understanding of survival patterns among septic ICU patients in Palestine. Finally, reliance on admission-only parameters may overlook dynamic clinical changes. Nonetheless, early physiologic and laboratory measures remain indispensable for prompt triage and intervention in settings with limited continuous monitoring.

## Conclusion

This study represents the first comprehensive evaluation of sepsis mortality predictors in a Palestinian tertiary‑care ICU, addressing a critical regional evidence gap. We demonstrate that a constellation of readily obtainable variables, including advancing age, cardiovascular comorbidity, tachycardia, reduced urine output, elevated lactate, prolonged ventilator days, impaired oxygenation indices (PaO_2_ and PaO_2_/FiO_2_ ratio), and shorter ICU length of stay, independently stratify patients by in‑hospital mortality risk. Translating these insights into practice, embedding such predictors into admission checklists and electronic alerts could enable early identification of high‑risk cases, guiding timely hemodynamic optimization, respiratory support, and resource prioritization. Because these measures are universally available and reproducible, they offer a pragmatic framework for strengthening sepsis care in resource‑limited environments. Prospective, multicenter validation of this tailored risk‑stratification model is warranted to inform national ICU protocols, optimize allocation of scarce resources, and ultimately improve sepsis outcomes across comparable low‑resource healthcare systems.

## Supporting information

S1 Table Adherence to the STROBE statement (cohort studies). (DOCX)

S2 Table The data collection form. (DOCX)

S3 Table Culture findings, treatment, ventilation, and discharge outcomes (n = 326). (DOCX)

S4 Table Associations between discharge outcomes and the variables of the patients (n = 326). (DOCX)

S1 Fig Source of infection. (DOCX)

S2 Fig Glasgow Coma Scale scores of the patients. (DOCX)

S1 Data The raw data. (XLSX)

## Abbreviations

ICU: Intensive care unit
APACHE II: Acute Physiology and Chronic Health Evaluation II
CFU: Colony forming units
CRP: C-reactive protein
FiO_2_: Fraction of inspired oxygen ratio
GCS: Glasgow Coma Scale
IRB: Institutional Review Board
IQR: Interquartile range
MEWS: Modified early warning score
OR: Odds ratio
PaO_2_/FiO_2_: Partial pressure of oxygen/fraction of inspired oxygen ratio
Q1: Lower quartile
Q3: Upper quartile
SOFA: Sequential Organ Failure Assessment
SAPS II: Simplified Acute Physiology Score II
SE: Standard error
SPSS: Statistical Package of Social Science
STROBE: Strengthening the Reporting of Observational Studies in Epidemiology
VIF: Variance inflation factor.
