# Supplementary material for: Factors predicting discharge outcomes of sepsis patients admitted to intensive care unit in a major tertiary care hospital: A retrospective study from Palestine
Source: PLOS Glob Public Health. 2025 Dec 19;5(12):e0005643. doi: 10.1371/journal.pgph.0005643 (PMC12716785; doi:10.1371/journal.pgph.0005643)
Supplement: S1 Table — (DOCX) [file pgph.0005643.s001.docx]

**S1 Table**

Adherence to the STROBE statement (cohort studies)

|  | **#** | **Recommendation** | **Section** |
| --- | --- | --- | --- |
| **Title and abstract** | 1 | (*a*) Indicate the study’s design with a commonly used term in the title or the abstract | Title and abstract |
|  |  | (*b*) Provide in the abstract an informative and balanced summary of what was done and what was found | Abstract |
| **Introduction** |  |  |  |
| Background/rationale | 2 | Explain the scientific background and rationale for the investigation being reported | Introduction: 1^st^ and 2^nd^ paragraphs |
| Objectives | 3 | State specific objectives, including any prespecified hypotheses | Introduction: last paragraph |
| **Methods** |  |  |  |
| Study design | 4 | Present key elements of study design early in the paper | Methods: Study design |
| Setting | 5 | Describe the setting, locations, and relevant dates, including periods of recruitment, exposure, follow-up, and data collection | Methods: Setting |
| Participants | 6 | (*a*) *Cohort study*—Give the eligibility criteria, and the sources and methods of selection of participants. Describe methods of follow-up | Methods: Participants |
|  |  | (*b*) *Cohort study*—For matched studies, give matching criteria and number of exposed and unexposed | N/A |
| Variables | 7 | Clearly define all outcomes, exposures, predictors, potential confounders, and effect modifiers. Give diagnostic criteria, if applicable | Methods: Variables |
| Data sources/ measurement | 8* | For each variable of interest, give sources of data and details of methods of assessment (measurement). Describe comparability of assessment methods if there is more than one group | Methods: Data sources and collection |
| Bias | 9 | Describe any efforts to address potential sources of bias | Methods: Data sources and collection + Limitations |
| Study size | 10 | Explain how the study size was arrived at | Methods: Sample size |
| Quantitative variables | 11 | Explain how quantitative variables were handled in the analyses. If applicable, describe which groupings were chosen and why | Methods: Data sources and collection + Statistical methods |
| Statistical methods | 12 | (*a*) Describe all statistical methods, including those used to control for confounding | Methods: Statistical methods |
|  |  | (*b*) Describe any methods used to examine subgroups and interactions | Methods: Statistical methods |
|  |  | (*c*) Explain how missing data were addressed | Methods: Participants |
|  |  | (*d*) *Cohort study*—If applicable, explain how loss to follow-up was addressed | N/A |
|  |  | (*e*) Describe any sensitivity analyses | Methods: Statistical methods |
| **Results** |  |  |  |
| Participants | 13* | (a) Report numbers of individuals at each stage of study—eg numbers potentially eligible, examined for eligibility, confirmed eligible, included in the study, completing follow-up, and analyzed | Results: Demographic and baseline variables of the patients |
|  |  | (b) Give reasons for non-participation at each stage | Methods: Participants |
|  |  | (c) Consider use of a flow diagram | N/A |
| Descriptive data | 14* | (a) Give characteristics of study participants (eg demographic, clinical, social) and information on exposures and potential confounders | Results: Demographic and baseline variables of the patients |
|  |  | (b) Indicate number of participants with missing data for each variable of interest | Results: Demographic and baseline variables of the patients |
|  |  | (c) *Cohort study*—summarize follow-up time (eg, average and total amount) | Results: Demographic and baseline variables of the patients + Other sections + Tables |
| Outcome data | 15* | *Cohort study*—Report numbers of outcome events or summary measures over time | Results: Demographic and baseline variables of the patients + Other sections + Tables |
|  |  | *Case-control study—*Report numbers in each exposure category, or summary measures of exposure | Results: Demographic and baseline variables of the patients + Other sections + Tables |
|  |  | *Cross-sectional study—*Report numbers of outcome events or summary measures | Results: Demographic and baseline variables of the patients + Other sections + Tables |
| Main results | 16 | (*a*) Give unadjusted estimates and, if applicable, confounder-adjusted estimates and their precision (eg, 95% confidence interval). Make clear which confounders were adjusted for and why they were included | Results: Demographic and baseline variables of the patients + Other sections + Tables |
|  |  | (*b*) Report category boundaries when continuous variables were categorized | Results: Demographic and baseline variables of the patients + Other sections + Tables |
|  |  | (*c*) If relevant, consider translating estimates of relative risk into absolute risk for a meaningful time period | Results: Demographic and baseline variables of the patients + Other sections + Tables |
| Other analyses | 17 | Report other analyses done—eg analyses of subgroups and interactions, and sensitivity analyses | Results: Demographic and baseline variables of the patients + Other sections + Tables |
| **Discussion** |  |  |  |
| Key results | 18 | summarize key results with reference to study objectives | Discussion: 1^st^ paragraph |
| Limitations | 19 | Discuss limitations of the study, taking into account sources of potential bias or imprecision. Discuss both direction and magnitude of any potential bias | Discussion: Limitations |
| Interpretation | 20 | Give a cautious overall interpretation of results considering objectives, limitations, multiplicity of analyses, results from similar studies, and other relevant evidence | Discussion section |
| Generalizability | 21 | Discuss the generalizability (external validity) of the study results | Discussion: Limitations +Discussion |
| **Other information** |  |  |  |
| Funding | 22 | Give the source of funding and the role of the funders for the present study and, if applicable, for the original study on which the present article is based | Declarations |
