# Supplementary material for: Factors predicting discharge outcomes of sepsis patients admitted to intensive care unit in a major tertiary care hospital: A retrospective study from Palestine
Source: PLOS Glob Public Health. 2025 Dec 19;5(12):e0005643. doi: 10.1371/journal.pgph.0005643 (PMC12716785; doi:10.1371/journal.pgph.0005643)
Supplement: S2 Table — (DOCX) [file pgph.0005643.s002.docx]

**S2 Table**

The data collection form

| **Age in years** |  |  |
| --- | --- | --- |
| **Sex** | □ Male | □ Female |
| **Comorbidities** | Details: |  |
| **neurologic status** |  |  |
| Unresponsive (GCS ≤ 8) | □ No | □ Yes |
| Verbal responsiveness (able to speak) | □ No | □ Yes |
| Pain responsiveness (able to report pain) | □ No | □ Yes |
| **Source of infection** | Details: |  |
| **Vital signs** |  |  |
| Systolic blood pressure (mmHg) |  |  |
| Diastolic blood pressure (mmHg) |  |  |
| Mean arterial pressure (mmHg) |  |  |
| Heart rate (beats/min) |  |  |
| Temperature (°C) |  |  |
| Respiratory rate (breaths/min) |  |  |
| MEWS |  |  |
| GCS |  |  |
| **Laboratory findings** |  |  |
| Urine output over 24 hours (mL/day) |  |  |
| CRP (mg/L) |  |  |
| Bicarbonate (mmol/L) |  |  |
| Platelet count (K/uL) |  |  |
| Total serum bilirubin (mg/dL) |  |  |
| PaO_2_ (mmHg) |  |  |
| Hematocrit (%) |  |  |
| White blood cells (K/uL) |  |  |
| Serum creatinine (mg/dL) |  |  |
| Blood urea nitrogen (mg/dL) |  |  |
| Serum sodium (mmol/L) |  |  |
| Serum potassium (mmol/L) |  |  |
| pH |  |  |
| Serum albumin (g/dL) |  |  |
| Serum lactate (mmol/L) |  |  |
| **Culture findings** |  |  |
| Blood | □ Negative | □ Positive |
| Urine | □ Negative | □ Positive |
| Tracheal aspirate culture | □ Negative | □ Positive |
| Sputum | □ Negative | □ Positive |
| **Antibiotics** | Details: |  |
| **Vasoactive agents** | Details: |  |
| **Mechanical ventilation** |  |  |
| Invasive ventilation | □ No | □ Yes |
| Noninvasive ventilation | □ No | □ Yes |
| PaO_2_ |  |  |
| Ventilator (Days) |  |  |
| PaO_2_/FiO_2_ |  |  |
| APACHE II (score) |  |  |
| SOFA (score) |  |  |
| SAPS II (score) |  |  |
| Length of stay (days) |  |  |
| **Discharge outcomes** | □ Alive | □ Dead |

APACHE II: Acute Physiology and Chronic Health Evaluation II, CRP: C-reactive protein, GCS: Glasgow Coma Scale, MEWS: Modified early warning score, PaO_2_: Fraction of inspired oxygen, FiO_2_: Fraction of inspired oxygen ratio, PaO_2_/FiO_2_: Partial pressure of oxygen/fraction of inspired oxygen ratio, SOFA: Sequential Organ Failure Assessment, SAPS II: Simplified Acute Physiology Score II. Note: Demographic characteristics, comorbidities, vital signs, laboratory parameters, and treatment exposures were collected a priori. Severity scores (APACHE II, SOFA, SAPS II), predicted mortality probabilities, and derived indices such as PaO_2_/FiO_2_ ratio were calculated post hoc based on recorded clinical and laboratory values. This distinction ensured that core predictors were prospectively defined, while derived metrics were computed using validated scoring algorithms.
