# Supplementary material for: Factors predicting discharge outcomes of sepsis patients admitted to intensive care unit in a major tertiary care hospital: A retrospective study from Palestine
Source: PLOS Glob Public Health. 2025 Dec 19;5(12):e0005643. doi: 10.1371/journal.pgph.0005643 (PMC12716785; doi:10.1371/journal.pgph.0005643)
Supplement: S3 Table — (DOCX) [file pgph.0005643.s003.docx]

**S3 Table**

Culture findings, treatment, ventilation, and discharge outcomes (*n = 326*)

| **Variable** | **n (%) or Median [Q1, Q3]** |
| --- | --- |
| **Culture findings** |  |
| **Blood** |  |
| Negative, n (%) | 263 (80.7) |
| Positive, n (%) | 63 (19.3) |
| **Urine** |  |
| Negative, n (%) | 267 (81.9) |
| Positive, n (%) | 59 (18.1) |
| **Tracheal aspirate culture** |  |
| Negative, n (%) | 289 (88.7) |
| Positive, n (%) | 37 (11.3) |
| **Sputum** |  |
| Negative, n (%) | 285 (87.4) |
| Positive, n (%) | 41 (12.6) |
| **Antibiotics** |  |
| Vancomycin, n (%) | 203 (62.3) |
| Meropenem, n (%) | 185 (56.7) |
| Levofloxacin, n (%) | 79 (24.2) |
| Piperacillin/tazobactam, n (%) | 68 (20.9) |
| Colistin, n (%) | 60 (18.4) |
| Tigecycline, n (%) | 54 (16.6) |
| Amikacin, n (%) | 41 (12.6) |
| Ciprofloxacin, n (%) | 11 (3.4) |
| Ceftazidime, n (%) | 10 (3.1) |
| Gentamicin, n (%) | 10 (3.1) |
| Trimethoprim/sulfamethoxazole, n (%) | 5 (1.5) |
| **Vasoactive agents** |  |
| Norepinephrine, n (%) | 237 (72.7) |
| Epinephrine, n (%) | 14 (4.3) |
| Dobutamine, n (%) | 2 (0.6) |
| Dopamine, n (%) | 1 (0.3) |
| **Mechanical ventilation** |  |
| Invasive ventilation |  |
| No, n (%) | 193 (59.2) |
| Yes, n (%) | 133 (40.8) |
| **Noninvasive ventilation** |  |
| No, n (%) | 140 (42.9) |
| Yes, n (%) | 186 (57.1) |
| PaO_2_, Median [Q1, Q3] | 4.0 [0.3, 180.0] |
| Ventilator (Days), Median [Q1, Q3] | 8.0 [0.4, 255.5] |
| PaO_2_/FiO_2_, Median [Q1, Q3] | 19.8 [0.5, 340.5] |
| APACHE II (score), Median [Q1, Q3] | 19.0 [15.0, 27.0] |
| SOFA (score), Median [Q1, Q3] | 9.0 [6.0, 12.0] |
| SAPS II (score), Median [Q1, Q3] | 47.0 [31.0, 63.0] |
| Length of stay (days), Median [Q1, Q3] | 8.0 [4.0, 16.0] |
| **Discharge outcomes** |  |
| Discharged alive | 191 (58.6%) |
| Discharged dead | 135 (41.4%) |

APACHE II: Acute Physiology and Chronic Health Evaluation II, CRP: C-reactive protein, GCS: Glasgow Coma Scale, MEWS: Modified early warning score, PaO_2_: fraction of inspired oxygen, PaO_2_/FiO_2_: Partial pressure of oxygen/fraction of inspired oxygen ratio, SOFA: Sequential Organ Failure Assessment, SAPS II: Simplified Acute Physiology Score II, Q1: lower quartile, Q3: upper quartile, statistically significant p-values are in boldface. Note: All variables were analyzed in the full cohort of 326 patients (100%). Patients with incomplete records were excluded during data preparation; therefore, each variable reflects data from all included participants. For continuous variables, values are presented as median [IQR]; for categorical variables, counts are shown as n (%).
