# Supplementary material for: Factors predicting discharge outcomes of sepsis patients admitted to intensive care unit in a major tertiary care hospital: A retrospective study from Palestine
Source: PLOS Glob Public Health. 2025 Dec 19;5(12):e0005643. doi: 10.1371/journal.pgph.0005643 (PMC12716785; doi:10.1371/journal.pgph.0005643)
Supplement: S4 Table — (DOCX) [file pgph.0005643.s004.docx]

**S4 Table**

Associations between discharge outcomes and the variables of the patients (*n = 326*)

|  | **Discharge outcomes** | |  |
| --- | --- | --- | --- |
|  | **Discharged alive** | **Discharged dead** |  |
| **Variable** | **Median [Q1, Q3] or n (%)** | **Median [Q1, Q3] or n (%)** | **p-value** |
| Age (years), Median [Q1, Q3] | 57.0 [45.5, 68.0] | 59.0 [45.0, 68.0] | 0.389 |
| **Sex** |  |  |  |
| Male, n (%) | 108 (33.1) | 84 (25.8) | 0.305 |
| Female, n (%) | 83 (25.5) | 51 (15.6) |  |
| **Comorbidities** |  |  |  |
| **Hypertension** |  |  |  |
| No, n (%) | 102 (31.3) | 92 (28.2) | **0.008** |
| Yes, n (%) | 89 (27.3) | 43 (13.2) |  |
| **Malignancy** |  |  |  |
| No, n (%) | 99 (30.4) | 36 (11.0) | **< 0.001** |
| Yes, n (%) | 92 (28.2) | 99 (30.4) |  |
| **Cardiovascular disease** |  |  |  |
| No, n (%) | 131 (40.2) | 110 (33.7) | **0.009** |
| Yes, n (%) | 60 (18.4) | 25 (7.7) |  |
| **Systemic inflammatory response syndrome** |  |  |  |
| No, n (%) | 64 (19.6) | 30 (9.2) | **0.027** |
| Yes, n (%) | 127 (39.0) | 105 (32.2) |  |
| **Diabetes mellitus** |  |  |  |
| No, n (%) | 120 (36.8) | 98 (30.1) | 0.065 |
| Yes, n (%) | 71 (21.8) | 37 (11.3) |  |
| **Renal disease** |  |  |  |
| No, n (%) | 119 (36.5) | 87 (26.7) | 0.693 |
| Yes, n (%) | 72 (22.1) | 48 (14.7) |  |
| **Liver disease** |  |  |  |
| No, n (%) | 176 (54.0) | 121 (37.1) | 0.432 |
| Yes, n (%) | 15 (4.6) | 14 (4.3) |  |
| **Mental status** |  |  |  |
| **Unresponsiveness** |  |  |  |
| No, n (%) | 155 (47.5) | 66 (20.2) | **< 0.001** |
| Yes, n (%) | 36 (11.0) | 69 (21.2) |  |
| **Verbal communication** |  |  |  |
| No, n (%) | 189 (58.0) | 122 (37.4) | **< 0.001** |
| Yes, n (%) | 2 (0.6) | 13 (4.0) |  |
| **Able to report pain** |  |  |  |
| No, n (%) | 188 (57.7) | 132 (40.5) | 0.695 |
| Yes, n (%) | 3 (0.9) | 3 (0.9) |  |
| **Vital signs** |  |  |  |
| Systolic blood pressure (mmHg), Median [Q1, Q3] | 110.0 [100.0, 124.5] | 103.0 [90.0, 115.0] | **< 0.001** |
| Diastolic blood pressure (mmHg), Median [Q1, Q3] | 60.0 [50.0, 70.0] | 60.0 [50.0, 70.0] | 0.673 |
| Mean arterial pressure (mmHg), Median [Q1, Q3] | 76.0 [69.5, 86.0] | 74.0 [66.0, 83.0] | **0.039** |
| Heart rate (beats/min), Median [Q1, Q3] | 96.0 [80.0, 111.0] | 110.0 [100.0, 120.0] | **< 0.001** |
| Temperature (°C), Median [Q1, Q3] | 36.9 [36.5, 37.7] | 37.0 [36.5, 37.9] | 0.473 |
| Respiratory rate (breaths/min), Median [Q1, Q3] | 23.0 [19.0, 25.0] | 24.0 [20.0, 28.0] | 0.081 |
| MEWS, Median [Q1, Q3] | 4.0 [2.0, 5.0] | 6.0 [4.0, 7.0] | **< 0.001** |
| GCS, Median [Q1, Q3] | 15.0 [14.0, 15.0] | 3.0 [3.0, 14.0] | **< 0.001** |
| **Laboratory findings** |  |  |  |
| Urine output over 24 hours (mL/day), Median [Q1, Q3] | 1895.0 [980.0, 2580.0] | 1440.0 [800.0, 2350.0] | **0.042** |
| CRP (mg/L), Median [Q1, Q3] | 136.0 [64.5, 253.0] | 187.0 [96.5, 265.5] | **0.035** |
| Platelet count (K/uL), Median [Q1, Q3] | 179.0 [67.5, 263.0] | 94.0 [26.0, 256.5] | **0.004** |
| Total serum bilirubin (mg/dL), Median [Q1, Q3] | 0.7 [0.4, 1.6] | 1.1 [0.5, 3.3] | **< 0.001** |
| Serum sodium (mmol/L), Median [Q1, Q3] | 137.0 [134.5, 141.0] | 140.0 [136.0, 144.0] | **< 0.001** |
| pH, Median [Q1, Q3] | 7.4 [7.3, 7.4] | 7.3 [7.3, 7.4] | **0.003** |
| Serum albumin (g/dL), Median [Q1, Q3] | 2.8 [2.5, 3.1] | 2.6 [2.2, 3.0] | **< 0.001** |
| Serum lactate (mmol/L), Median [Q1, Q3] | 1.6 [1.1, 2.9] | 2.8 [1.7, 4.6] | **< 0.001** |
| Bicarbonate (mmol/L), Median [Q1, Q3] | 22.0 [19.0, 25.0] | 20.6 [17.0, 25.0] | 0.073 |
| PaO_2_ (mmHg), Median [Q1, Q3] | 97.0 [80.5, 119.0] | 99.0 [80.5, 120.0] | 0.916 |
| Hematocrit (%), Median [Q1, Q3] | 27.2 [23.3, 31.6] | 26.2 [22.9, 29.3] | 0.091 |
| White blood cells (K/uL), Median [Q1, Q3] | 10.6 [6.4, 17.0] | 10.5 [5.2, 18.9] | 0.799 |
| Serum creatinine (mg/dL), Median [Q1, Q3] | 1.1 [0.7, 3.5] | 1.3 [0.6, 2.7] | 0.650 |
| Blood urea nitrogen (mg/dL), Median [Q1, Q3] | 28.0 [16.0, 57.6] | 32.0 [19.0, 54.0] | 0.366 |
| Serum potassium (mmol/L), Median [Q1, Q3] | 4.1 [3.7, 4.6] | 4.1 [3.8, 4.7] | 0.270 |
| **Culture findings and antibiotics** |  |  |  |
| **Culture findings** |  |  |  |
| **Blood** |  |  |  |
| Negative, n (%) | 159 (48.8) | 104 (31.9) | 0.162 |
| Positive, n (%) | 32 (9.8) | 31 (9.5) |  |
| **Urine** |  |  |  |
| Negative, n (%) | 157 (48.2) | 110 (33.7) | 0.868 |
| Positive, n (%) | 34 (10.4) | 25 (7.7) |  |
| **Tracheal aspirate culture** |  |  |  |
| Negative, n (%) | 173 (53.1) | 116 (35.6) | 0.192 |
| Positive, n (%) | 18 (5.5) | 19 (5.8) |  |
| **Sputum** |  |  |  |
| Negative, n (%) | 166 (50.9) | 119 (36.5) | 0.740 |
| Positive, n (%) | 25 (7.7) | 16 (4.9) |  |
| **Treatment** |  |  |  |
| **Levofloxacin** |  |  |  |
| No, n (%) | 134 (41.1) | 113 (34.7) | **0.005** |
| Yes, n (%) | 57 (17.5) | 22 (6.7) |  |
| **Tigecycline** |  |  |  |
| No, n (%) | 168 (51.5) | 104 (31.9) | **0.009** |
| Yes, n (%) | 23 (7.1) | 31 (9.5) |  |
| **Vasoactive agents** |  |  |  |
| No, n (%) | 69 (21.2) | 19 (5.8) | **< 0.001** |
| Yes, n (%) | 122 (37.4) | 116 (35.6) |  |
| **Vancomycin** |  |  |  |
| No, n (%) | 74 (22.7) | 49 (15.0) | 0.653 |
| Yes, n (%) | 117 (35.9) | 86 (26.4) |  |
| **Meropenem** |  |  |  |
| No, n (%) | 87 (26.7) | 54 (16.6) | 0.319 |
| Yes, n (%) | 104 (31.9) | 81 (24.8) |  |
| **Piperacillin/tazobactam** |  |  |  |
| No, n (%) | 147 (45.1) | 111 (34.0) | 0.250 |
| Yes, n (%) | 44 (13.5) | 24 (7.4) |  |
| **Colistin** |  |  |  |
| No, n (%) | 161 (49.4) | 105 (32.2) | 0.135 |
| Yes, n (%) | 30 (9.2) | 30 (9.2) |  |
| **Amikacin** |  |  |  |
| No, n (%) | 164 (50.3) | 121 (37.1) | 0.312 |
| Yes, n (%) | 27 (8.3) | 14 (4.3) |  |
| **Ciprofloxacin** |  |  |  |
| No, n (%) | 185 (56.7) | 130 (39.9) | 0.782 |
| Yes, n (%) | 6 (1.8) | 5 (1.5) |  |
| **Ceftazidime** |  |  |  |
| No, n (%) | 186 (57.1) | 130 (39.9) | 0.575 |
| Yes, n (%) | 5 (1.5) | 5 (1.5) |  |
| **Gentamicin** |  |  |  |
| No, n (%) | 186 (57.1) | 130 (39.9) | 0.575 |
| Yes, n (%) | 5 (1.5) | 5 (1.5) |  |
| **Trimethoprim/sulfamethoxazole** |  |  |  |
| No, n (%) | 188 (57.7) | 133 (40.8) | 0.949 |
| Yes, n (%) | 3 (0.9) | 2 (0.6) |  |
| **Mechanical ventilation** |  |  |  |
| **Invasive ventilation** |  |  |  |
| No, n (%) | 150 (46.0) | 43 (13.2) | **< 0.001** |
| Yes, n (%) | 41 (12.6) | 92 (28.2) |  |
| Ventilator (Days), Median [Q1, Q3] | 8.5 [5.0, 19.0] | 8.0 [3.0, 21.0] | 0.557 |
| Length of stay (Days), Median [Q1, Q3] | 8.0 [4.0, 14.0] | 8.0 [3.0, 19.5] | 0.936 |
| **Noninvasive ventilation** |  |  |  |
| No, n (%) | 46 (14.1) | 94 (28.8) | **< 0.001** |
| Yes, n (%) | 145 (44.5) | 41 (12.6) |  |
| FiO_2_, Median [Q1, Q3] | 0.4 [0.3, 0.5] | 0.5 [0.4, 0.6] | **< 0.001** |
| PaO2/FiO_2_, Median [Q1, Q3] | 296.0 [212.5, 360.5] | 216.0 [151.0, 284.3] | **< 0.001** |
| APACHE II (score), Median [Q1, Q3] | 16.0 [12.5, 23.0] | 26.0 [19.0, 31.0] | **< 0.001** |
| SOFA (score), Median [Q1, Q3] | 8.0 [5.0, 10.0] | 12.0 [9.0, 14.0] | **< 0.001** |
| SAPS II (score), Median [Q1, Q3] | 36.0 [27.0, 51.0] | 60.0 [48.0, 72.5] | **< 0.001** |

APACHE II: Acute Physiology and Chronic Health Evaluation II, CRP: C-reactive protein, GCS: Glasgow Coma Scale, MEWS: Modified early warning score, PaO_2_: Fraction of inspired oxygen, FiO_2_: Fraction of inspired oxygen ratio, PaO_2_/FiO_2_: Partial pressure of oxygen/fraction of inspired oxygen ratio, SOFA: Sequential Organ Failure Assessment, SAPS II: Simplified Acute Physiology Score II, Q1: Lower quartile, Q3: Upper quartile, statistically significant p-values are in boldface. Note: All variables were analyzed in the full cohort of 326 patients (100%). Patients with incomplete records were excluded during data preparation; therefore, each variable reflects data from all included participants. For continuous variables, values are presented as median [IQR]; for categorical variables, counts are shown as n (%). Invasive and non‑invasive ventilation were coded as separate binary variables. Totals therefore represent independent counts and are not mutually exclusive categories. Patients may appear in one group only (invasive or non‑invasive) or in neither, depending on the modality received.
