# Supplementary material for: Factors predicting discharge outcomes of sepsis patients admitted to intensive care unit in a major tertiary care hospital: A retrospective study from Palestine
Source: PLOS Glob Public Health. 2025 Dec 19;5(12):e0005643. doi: 10.1371/journal.pgph.0005643 (PMC12716785; doi:10.1371/journal.pgph.0005643)
Supplement: S2 Fig — (DOCX) [file pgph.0005643.s006.docx]

**S2 Fig**

Glasgow Coma Scale scores of the patients
